# Supplementary material for: Expansion of the circadian transcriptome in Brassica rapa and genome-wide diversification of paralog expression patterns
Source: eLife. 2020 Sep 30;9:e58993. doi: 10.7554/eLife.58993 (PMC7655105; doi:10.7554/eLife.58993)
Supplement: Supplementary file 8. — DiPALM was evaluated using simulated benchmark datasets (Spies et al., 2019). Eight different simulated datasets were generated (rows). Dataset names denote the number of simulated counts, number of simulated replicates, and the number of simulated timepoints. This table also includes the top three performing methods mentioned in Spies et al.: splineTC (Michna et al., 2016), maSigPro (Nueda et al., 2014), and impulseDE2 (Fischer et al., 2018). The method of running a traditional differential expression analysis on each timepoint separately and then taking the most significant timepoint for each gene is also shown (pairwise). Three different DiPALM evaluations were carried out using the pattern-detecting version (kME), the abundance-detecting version (Median) and a combined score using the sum of both. [file elife-58993-supp8.docx]

**Supplemental Table**

|  | **DiPALM**  **kME** | **DiPALM**  **Median** | **DiPALM**  **Combine** | **Pairwise** | **splineTC** | **maSigPro** | **impulseDE2** |
| --- | --- | --- | --- | --- | --- | --- | --- |
| **10M_3rep_4TP** | 0.902 | 0.923 | 0.937 | 0.974 | 0.939 | 0.833 | 0.939 |
| **20M_3rep_4TP** | 0.908 | 0.925 | 0.94 | 0.979 | 0.939 | 0.832 | 0.949 |
| **30M_2rep_4TP** | 0.863 | 0.905 | 0.899 | 0.967 | 0.915 | 0.83 | 0.927 |
| **30M_3rep_12TP** | 0.525 | 0.905 | 0.597 | 0.915 | 0.869 | 0.867 | 0.93 |
| **30M_3rep_4TP** | 0.905 | 0.926 | 0.941 | 0.98 | 0.939 | 0.831 | 0.952 |
| **30M_3rep_8TP** | 0.955 | 0.951 | 0.962 | 0.987 | 0.966 | 0.882 | 0.979 |
| **30M_5rep_4TP** | 0.953 | 0.941 | 0.968 | 0.989 | 0.965 | 0.802 | 0.972 |
| **50M_3rep_4TP** | 0.908 | 0.926 | 0.936 | 0.98 | 0.939 | 0.83 | 0.953 |

**Table S1 DiPALM Benchmarking Using The Area Under the Receiver Operating Characteristic  (AUROC) Curve.** DiPALM was evaluated using a published, simulated benchmark datasets (Spies et al., 2017). Eight different simulated datasets were generated (rows). Dataset names denote the number of simulated counts, number of simulated replicates and the number of simulated timepoints. This table also includes the top 3 performing methods mentioned in Spies et al.: splineTC (Michna et al., 2016), maSigPro (Nueda et al., 2014) and impulseDE2 (Fischer et al., 2018). The method of running a traditional differential expression analysis on each timepoint separately and then taking the most significant timepoint for each gene is also shown (pairwise). Three different DiPALM evaluations were carried out using the pattern-detecting version (kME), the abundance-detecting version (Median) and a combined score using the sum of both.
