# Supplementary material for: Expansion of the circadian transcriptome in Brassica rapa and genome-wide diversification of paralog expression patterns
Source: eLife. 2020 Sep 30;9:e58993. doi: 10.7554/eLife.58993 (PMC7655105; doi:10.7554/eLife.58993)
Supplement: Transparent reporting form [file elife-58993-transrepform.docx]

***eLife’s* transparent reporting form**

We encourage authors to provide detailed information *within their submission* to facilitate the interpretation and replication of experiments. Authors can upload supporting documentation to indicate the use of appropriate reporting guidelines for health-related research (see [EQUATOR Network](about:blank)), life science research (see the [BioSharing Information Resource](about:blank)), or the [ARRIVE guidelines](about:blank) for reporting work involving animal research. Where applicable, authors should refer to any relevant reporting standards documents in this form.

If you have any questions, please consult our Journal Policies and/or contact us: [editorial@elifesciences.org](about:blank).

**Sample-size estimation**

- You should state whether an appropriate sample size was computed when the study was being designed
- You should state the statistical method of sample size computation and any required assumptions
- If no explicit power analysis was used, you should describe how you decided what sample (replicate) size (number) to use

Please outline where this information can be found within the submission (e.g., sections or figure legends), or explain why this information doesn’t apply to your submission:

For circadian RNA-seq time course experiments, the number of sequence libraries generated was determined by the cost associated with RNA sequencing. We chose the highest possible sampling density (2 hours) that allowed for replication.

**Replicates**

- You should report how often each experiment was performed
- You should include a definition of biological versus technical replication
- The data obtained should be provided and sufficient information should be provided to indicate the number of independent biological and/or technical replicates
- If you encountered any outliers, you should describe how these were handled
- Criteria for exclusion/inclusion of data should be clearly stated
- High-throughput sequence data should be uploaded before submission, with a private link for reviewers provided (these are available from both GEO and ArrayExpress)

Please outline where this information can be found within the submission (e.g., sections or figure legends), or explain why this information doesn’t apply to your submission:

The 2-hour sampling density of the time course data allowed for 2 biological replicates. Experimental details are provided in the ‘Materials and Methods’ section under ‘Circadian Transcriptome Growth Conditions’. Data is already publicly available under GEO accession GSE123654, as indicated in the ‘Materials and Methods’ section under ‘RNA-sequencing Library Preparations and Processing’ as well as in the ‘Availability of Data and Materials’ section.

**Statistical reporting**

- Statistical analysis methods should be described and justified
- Raw data should be presented in figures whenever informative to do so (typically when N per group is less than 10)
- For each experiment, you should identify the statistical tests used, exact values of N, definitions of center, methods of multiple test correction, and dispersion and precision measures (e.g., mean, median, SD, SEM, confidence intervals; and, for the major substantive results, a measure of effect size (e.g., Pearson's r, Cohen's d)
- Report exact p-values wherever possible alongside the summary statistics and 95% confidence intervals. These should be reported for all key questions and not only when the p-value is less than 0.05.

Please outline where this information can be found within the submission (e.g., sections or figure legends), or explain why this information doesn’t apply to your submission:

This manuscript introduces and utilizes a novel statistical analysis called Differential Pattern Analysis via Linear Models (DiPALM) that assigns statistical significance to genes based on pattern changes between two or more experimental conditions. This analysis has been built as an R package available through the CRAN repository. Source code and details about the methods and usage can be found in the package documentation and vignette.

All analyses performed in this manuscript are described and documented in R source code and available through the Greenham lab github repository (see ‘Bioinformatic and Statistical Analysis’ section of materials and methods). This repository contains files with input data into the analysis as well as full documentation via an R markdown file (Brapa_CircadianTranscriptome_Markdown.Rmd). Below are the major statistical analyses performed in the manuscript and the associated locations in the above .Rmd file.

- [Line 357] DiPALM analysis of LDHH vs LLHC entrainment conditions.

- [Line 438] Comparison of expression levels for genes with retained paralogs vs. single genes. Using one-way ANOVA and Tukey test.

- [Line 535] Enrichment and Depletion of copied genes in morning and evening phases. Using hypergeometric test

- [Line 600] DiPALM analysis of paralogs in combined LD and HC datasets.

- [Line 943] Identify the arabidopsis-like *B. rapa* paralog. Using a custom permutation test where same-sized At, Br1 and Br2 target groups are randomly generated and tested 10,000 times then compared to the observed groups.

- [Line 1053] Similar analysis repeated to determine the more arabidopsis-like *B. rapa* paralog based on target CNS sequences.

- [Line 1312] DiPALM analysis of paralogs under diel conditions

- [Line 1394] DiPALM analysis of drought response

- [Line 1480] Test for over-representation of drought-responsive genes in the set of paralog pairs. Using a custom random sampling analysis to generate a distribution of 10,000 null tests.

- [Line 1511] Test for significance of one and/or both paralogs being drought-responsive. Using the same null distribution from above to test for 1 or both paralogs being drought responsive.

(For large datasets, or papers with a very large number of statistical tests, you may upload a single table file with tests, Ns, etc., with reference to sections in the manuscript.)

**Group allocation**

- Indicate how samples were allocated into experimental groups (in the case of clinical studies, please specify allocation to treatment method); if randomization was used, please also state if restricted randomization was applied
- Indicate if masking was used during group allocation, data collection and/or data analysis

Please outline where this information can be found within the submission (e.g., sections or figure legends), or explain why this information doesn’t apply to your submission:

All groups used for statistical testing were simple ‘Experiment vs. Control’ or ‘Paralog1 vs Paralog2’ groups. For paralog comparisons of expression levels, the paralog the highest expression was denoted as ‘Paralog 1’. For network-based comparisons between arabidopsis and *B. rapa*, the most arabidopsis-like paralog was denoted as ‘Paralog 1’.

**Additional data files (“source data”)**

- We encourage you to upload relevant additional data files, such as numerical data that are represented as a graph in a figure, or as a summary table
- Where provided, these should be in the most useful format, and they can be uploaded as “Source data” files linked to a main figure or table
- Include model definition files including the full list of parameters used
- Include code used for data analysis (e.g., R, MatLab)
- Avoid stating that data files are “available upon request”

Please indicate the figures or tables for which source data files have been provided:

All raw data are available through the two included GEO repository links found in the ‘Availability of Data and Materials’ section.

All additional source data as well as annotated source code for the entire analysis is available through the Greenham lab github repository found in the ‘Bioinformatic and Statistical Analysis’ section of materials and methods.
